# Genome wide analysis of DNA copy number neutral loss of heterozygosity (CNNLOH) and its relation to gene expression in esophageal squamous cell carcinoma

**DOI:** 10.1186/1471-2164-11-576

**Published:** 2010-10-18

**Authors:** Nan Hu, Robert J Clifford, Howard H Yang, Chaoyu Wang, Alisa M Goldstein, Ti Ding, Philip R Taylor, Maxwell P Lee

**Affiliations:** 1 Genetic Epidemiology Branch, Division of Cancer Epidemiology and Genetics, National Cancer Institute, NIH, DHHS, Bethesda, Maryland, USA; 2 Laboratory of Population Genetics, Center for Cancer Research, National Cancer Institute, NIH, DHHS, Bethesda, Maryland, USA; 3 Shanxi Cancer Hospital, Taiyuan, Shanxi, 030013, PR China

## Abstract

**Background:**

Genomic instability plays an important role in human cancers. We previously characterized genomic instability in esophageal squamous cell carcinomas (ESCC) in terms of loss of heterozygosity (LOH) and copy number (CN) changes in tumors using the Affymetrix GeneChip Human Mapping 500K array in 30 cases from a high-risk region of China. In the current study we focused on copy number neutral (CN = 2) LOH (CNNLOH) and its relation to gene expression in ESCC.

**Results:**

Overall we found that 70% of all LOH observed was CNNLOH. Ninety percent of ESCCs showed CNNLOH (median frequency in cases = 60%) and this was the most common type of LOH in two-thirds of cases. CNNLOH occurred on all 39 autosomal chromosome arms, with highest frequencies on 19p (100%), 5p (96%), 2p (95%), and 20q (95%). In contrast, LOH with CN loss represented 19% of all LOH, occurred in just half of ESCCs (median frequency in cases = 0%), and was most frequent on 3p (56%), 5q (47%), and 21q (41%). LOH with CN gain was 11% of all LOH, occurred in 93% of ESCCs (median frequency in cases = 13%), and was most common on 20p (82%), 8q (74%), and 3q (42%). To examine the effect of genomic instability on gene expression, we evaluated RNA profiles from 17 pairs of matched normal and tumor samples (a subset of the 30 ESCCs) using Affymetrix U133A 2.0 arrays. In CN neutral regions, expression of 168 genes (containing 1976 SNPs) differed significantly in tumors with LOH versus tumors without LOH, including 101 genes that were up-regulated and 67 that were down-regulated.

**Conclusion:**

Our results indicate that CNNLOH has a profound impact on gene expression in ESCC, which in turn may affect tumor development.

## Background

Genomic instability is important for cancer development and can manifest as copy number (CN) gain or loss as well as loss of heterozygosity (LOH). Copy number neutral LOH (CNNLOH) has been observed in tumors following the widespread application of SNP array technology [1,2]. CNNLOH is common in many tumor types, including basal cell carcinoma [3], acute myeloid leukemia [4,5], medulloblastoma [6], melanoma [7], follicular lymphoma [8], colorectal cancers [9-11], glioblastoma [12,13], cutaneous squamous cell carcinomas [14], acute promyelocytic leukemia [15], acute lymphoblastic leukemia [16], ovarian tumor [17], and esophageal adenocarcinoma [18], and has recently been reviewed for myeloid malignancies [19]. CNNLOH is thought to result from mitotic recombination or nondisjunction in somatic tumor cells [3]. However, the distribution of complex DNA alterations and its relation to gene expression in tumors have not been characterized in ESCC.

ESCC is a common malignancy worldwide and one of the most common cancers in the Chinese population; Shanxi Province in north central China has some of the highest esophageal cancer rates in the world [20,21]. Previously, we identified several regions of LOH and CN alteration in ESCC using microsatellite markers and low- and high-density SNP arrays [22-27], where the majority of ESCC patients from this high-risk population were found to have high genomic instability and high frequency of LOH on several chromosome arms. However, we have not found causal mutations in candidate genes within the LOH regions identified. For example, 82% of 56 ESCCs showed LOH when tested with four microsatellite markers flanking *ANXA1 (*9q11-q21), but no somatic mutations were detected in these patients [28]. Another example is *BRCA2*, which also showed frequent LOH in ESCC (57% for D13S260, 83% for D13S767), but only infrequent somatic mutations in these cancer patients (2/56, 3.5%) [29,30]. Contrary to expectation, expression of *BRCA2 *was often increased (unpublished data).

In the present study, we analyzed DNA from 30 micro-dissected ESCC tumors, adjacent normal tissue, and blood DNA from the same patient using the Affymetrix 500K SNP array to identify the distribution of complex DNA alterations, including CNNLOH, and we related CNNLOH to expression of the genes affected as assessed with the Affymetrix U133A 2.0 array in these patients.

## Methods

### Case selection

This study was approved by the Institutional Review Boards of the Shanxi Cancer Hospital and the US National Cancer Institute (NCI). Cases diagnosed with ESCC between 1998 and 2001 in the Shanxi Cancer Hospital in Taiyuan, Shanxi Province, PR China, and considered candidates for curative surgical resection were identified and recruited to participate in this study. None of the cases had prior therapy and Shanxi was the ancestral home for all. After obtaining informed consent, cases were interviewed to obtain information on demographics, cancer risk factors (eg, smoking, alcohol drinking, and detailed family history of cancer), and clinical information. The cases evaluated here were part of a larger case-control study of upper gastrointestinal cancers conducted in Shanxi Province [31-33].

### Biological specimen collection and processing

Venous blood (10 ml) was taken from each case prior to surgery and germ-line DNA from whole blood was extracted and purified using the standard phenol/chloroform method.

Tumor and adjacent normal tissues were dissected at the time of surgery and stored in liquid nitrogen until used. One 5-micron section was H&E stained and reviewed by a pathologist from the NCI to guide the micro-dissection. Five to ten consecutive 8-micron sections were cut from fresh frozen tumor and adjacent normal tissues. Tumor and normal cells were manually micro-dissected under light microscopy. DNA was extracted from micro-dissected tumor as previously described [34] using the protocol from the Puregene DNA Purification Tissue Kit (Gentra Systems, Inc., Minneapolis, MN). RNA was extracted from 17 of these micro-dissected tumor and matched normal tissue pairs using the protocol from the PureLink Micro-to-Midi Total RNA Purification System (Catalog number 12183-018, Invitrogen, Carlsbad, CA). RNA quality and quantity were determined using the RNA 6000 Labchip/Agilent 2100 Bioanalyzer (Agilent Technologies, Germantown, MD). The same tissue blocks were used for extraction of both DNA and RNA for each case studied.

### Target preparation for GeneChip Human Mapping 500 K array set

The Affymetrix GeneChip Human Mapping 500 K array set contains ~262,000 (Nsp I array) and ~238,000 (Sty I array) SNPs (mean probe spacing = 5.8 Kb, mean heterozygosity = 27%). A detailed gene chip protocol can be found at http://www.affymetrix.com/support/downloads/manuals/500k_assay_manual.pdf.

Experiments were conducted according to the protocol (GeneChip Mapping Assay manual) supplied by Affymetrix, Inc. (Santa Clara, CA). Genotype calls were generated by GTYPE v 4.0 software (Affymetrix). Germ-line, tumor and adjacent normal DNA from each case were run together in parallel in the same experiment (ie, same batch, same day). The GEO accession numbers for these array data are GSE15526 and GSE20347.

### Probe preparation and hybridization for Human Genome U133A 2.0 array

The Affymetrix Human Genome U133A 2.0 array is a single array used to interrogate expression of 14,500 well-characterized human genes. Array experiments were performed using 1-5 μg total RNA each. We followed the protocol provided by the manufacturer to carry out reverse transcription, labeling, and hybridization.

### GeneChip 500 K array data analysis

Probe intensity data from Affymetrix 500 K SNP arrays were used to identify DNA alterations in the present study. To avoid gender-related issues, SNPs mapped to either the X or Y chromosome were excluded.

Copy number (CN) loss or gain was based on comparisons of either adjacent normal to germ-line DNA or tumor to germ-line DNA. Microarray data were first normalized using the gtype-probe set-genotype package included in Affymetrix Power Tools version 1.85. Each tumor sample was individually normalized via the BRLMM algorithm along with 99 blood samples. These blood samples were obtained from the 30 ESCC cases evaluated in the present study plus 69 healthy controls (age-, sex-, and region-matched to cases) who were all part of a larger case-control study of upper gastrointestinal cancers conducted in Shanxi Province (as noted above). Paired CN analysis was then performed on each sample using the Affymetrix Power Tools *paired-copy-number *workflow, which implements the Affymetrix Copy Number Analysis Tool (CNAT) algorithm. DNA obtained from the blood of each case served as the normal control; a sliding window of 100 kb was chosen to optimize the identification of extended regions of CN alteration (see http://www.affymetrix.com/support/technical/whitepapers/cnat_4_algorithm_whitepaper.pdf). The output of the CNAT program is CN state rather than an absolute CN prediction: normal CN corresponds to a state of 2; zero and 1 correspond to CN loss; and states 3 and 4 correspond to CN gain.

In the present study, we modified the method for identifying LOH used in our previous studies [26,27]. Here, LOH was determined using the Affymetrix Power Tools *copynumber-pipeline *program *paired-LOH *workflow. Input was *.CHP files generated with the *gtype-probeset-genotype *package as described above. Matched blood DNA served as the reference for LOH analysis for each tumor and normal adjacent sample.

### Combination of LOH and CN alterations

We defined six combinations of copy number state and LOH status. LOH positive loci may have CN loss (CN ≤ 1), be CN neutral (CNNLOH, CN = 2) or show CN gain (CN ≥ 3); Likewise, LOH negative loci may show CN loss, gain, or neutrality. LOH and CN segments for each tumor were defined independently for each sample as contiguous blocks of informative SNPs that possessed the same LOH and CN state. Endpoints of LOH/CN segments were defined by informative SNPs. Some uninformative SNPs were located between these LOH/CN segments; we considered these SNPs to have an undefined LOH/CN state (see Additional file 1/Figure S1). Segment sizes were empirically observed from the data.

### Comparison of CN status in DNA from blood versus micro-dissected adjacent normal tissue

DNA isolated from normal adjacent tissue is frequently used as a control in microarray experiments. In the present study we used DNA isolated from peripheral blood. We expected peripheral blood DNA to be a superior control for two reasons: first, unlike adjacent normal tissue, it is does not run the risk of being contaminated with tumor cells; second, adjacent normal tissue may actually be precancerous and contain genetic lesions. To examine whether blood DNA and adjacent normal esophageal DNA were equivalent controls, we compared copy number state calls for blood and normal adjacent from each of the 30 ESCC patients. We found that the two controls were equivalent: 99.29% to 99.99% of all copy number calls were identical. Overall, 99.96% of SNPs in blood and 99.93% in normal adjacent tissue were CN = 2 state.

### Human Genome U133A 2.0 array data analysis and relation between CNNLOH and mRNA expression

The Robust Multiarray Average (RMA) algorithm [35,36] implemented in Bioconductor in R http://www.bioconductor.org was used for background correction and normalization across all samples. For each sample log2 fold changes in gene expression were calculated by subtracting the adjacent normal RMA value from the corresponding tumor RMA value.

To determine whether any gene showed a difference in the tumor versus normal gene expression fold change that was dependent on LOH state, we performed the following steps: (i) First, genes assayed by the U133A microarray were mapped onto each LOHCN segment of each sample. Map locations of genes were taken from the Affymetrix version *na29 *microarray annotation file. Note that probe sets from the same gene may have different reference sequences which differ in their chromosomal locations. Also, not every gene will map to every sample - in a particular sample, a gene may map to a gap between LOHCN regions. (ii) Next, we identified genes for which at least two of the 17 ESCC samples with expression data were LOH negative and at least two samples were LOH positive. (iii) We then performed two-sided unpaired t-tests comparing the log2 fold changes for a probe set in LOH positive and LOH negative samples. A *P*-value < 0.01 was considered significant. (iv) Finally, SNPs on the 500 K microarray were mapped to the reference sequence for each expression probe set. Since probe sets from the same gene may have different reference sequences, they may differ in the number of SNPs assigned to them (Additional file 2/Figure S2).

## Results

In the present study we determined copy number and loss of heterozygosity (LOH) status in DNA isolated from germ-line and micro-dissected tumor and matched adjacent normal samples from 30 ESCC patients using the Affymetrix 500 K SNP array. The average genotype call rate was 96% (89-99%): the 250 K Nsp I array was 96% (90-98%) and 250 K Sty I array was 95% (89-99%). Genotype call rates were similar for all three tissue types examined. We first analyzed whether copy numbers were similar between DNAs from the two normal tissues: germ-line (blood) and micro-dissected adjacent normal samples. Our analysis indicated that DNA CN values were similar between the two normal tissues (Additional file 3 - Table S1), as expected. Our results indicate that germ-line DNA can be used as a normal control in studies of CN alteration; it is more readily available than matched adjacent normal tissue.

### Complex DNA alterations in ESCC

The distribution of DNA alterations in each of the 30 ESCC cases is summarized in Table 1 (with LOH) and in Additional file 4/Table S2 (without LOH). We divided genomic regions into three groups based on CN states: CN loss, neutral, and gain. We found that 50%, 90%, and 93% of cases showed LOH in the CN loss, neutral, and gain groups, respectively (Table 1). For each chromosome, we also calculated the percentage of SNPs involved in LOH for each group. They ranged between 20-57%, 7-100%, and 2-100% for the CN loss, neutral, and gain groups, respectively (Table 1). Our results suggest that LOH with CN neutral or gain are common phenomena in ESCC. For SNPs without LOH, we also calculated the percent of SNPs in each CN state; averages were 5%, 84%, and 11% for CN loss, neutral, and gain, respectively.

**Table 1:** LOH by copy number in ESCC cases by individual case (N = 30)

Case ID	Total no. informative SNPs with LOH	No. informative SNPs with LOH and CN = 1 (fraction)	No. informative SNPs with LOH and CN = 2 (fraction)	No. informative SNPs with LOH and CN = 3 or 4 (fraction)
*1	31,808	2,260 (0.07)	26,801 (0.84)	2,747 (0.09)
2	368	0 (0)	205 (0.56)	163 (0.44)
3	377	0 (0)	139 (0.37)	238 (0.63)
*4	36,175	2,093 (0.06)	27,655 (0.76)	6,427 (0.18)
*5	24	0 (0)	0 (0)	24 (1.00)
*6	14,751	210 (0.01)	12,661 (0.86)	1,880 (0.13)
*7	3,559	0 (0)	2,905 (0.82)	654 (0.18)
*8	2,408	4 (0)	2,217 (0.92)	187 (0.08)
*9	593	1 (0)	266 (0.45)	326 (0.55)
10	17,546	1,075 (0.06)	12,087 (0.69)	4,384 (0.25)
11	78,159	3,678 (0.05)	67,726 (0.87)	6,755 (0.09)
12	41	0 (0)	0 (0)	41 (1.00)
*13	6,113	209 (0.03)	5,154 (0.84)	750 (0.12)
14	13,498	4,084 (0.30)	9,190 (0.68)	224 (0.02)
15	3	0 (0)	3 (1.00)	0 (0)
16	1431	0 (0)	54 (0.04)	1,377 (0.96)
17	16,934	5,732 (0.34)	10,842 (0.64)	360 (0.02)
18	2,107	0 (0)	1,553 (0.74)	554 (0.26)
*19	0	0 (0)	0 (0)	0 (0)
*20	527	0 (0)	137 (0.26)	390 (0.74)
*21	19,954	62 (0)	16,332 (0.82)	3,560 (0.18)
*22	11,180	4,410 (0.39)	6,357 (0.57)	413 (0.04)
*23	14,523	5,939 (0.41)	5,953 (0.41)	2,631 (0.18)
24	15,773	5,672 (0.36)	9,354 (0.59)	747 (0.05)
*25	23,005	20 (0)	17,114 (0.74)	5,871 (0.26)
*26	37,691	21,495 (0.57)	15,229 (0.40)	967 (0.03)
27	12,120	4,500 (0.37)	6,299 (0.52)	1,321 (0.11)
28	494	0 (0)	37 (0.07)	457 (0.93)
*29	18,222	444 (0.02)	15,400 (0.85)	2,378 (0.13)
*30	42,499	16,432 (0.39)	25,394 (0.60)	673 (0.02)

No. cases with SNP LOH fraction = 0		15	3	2
Range (fraction SNPs with LOH)		0-0.57	0-1.00	0-1.00
Median (fraction SNPs with LOH)		0.00	0.60	0.13
Global average (fraction SNPs with LOH)		0.19	0.70	0.11

*Case also examined with Affymetrix U133A 2.0 chip

**Figure 1:**
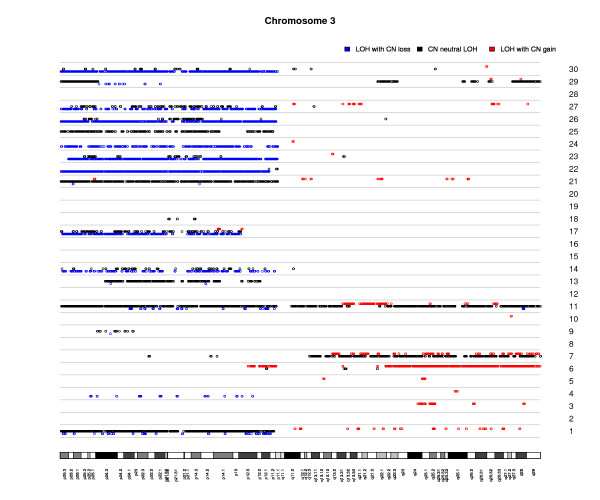
**Patterns of loss of heterozygosity and copy number variation in 30 ESCC samples for chromosome 3**. Each row (numbered 1 - 30) represents an individual ESCC sample. Circles indicate the positions of SNPs showing LOH. SNP positions are color coded as follows: **black **indicates copy number neutral LOH; **blue **indicates LOH accompanied by copy number reduction; **red **indicates LOH with copy number gain. An ideogram of the chromosome is at the bottom of the figure.

The distribution of the six types of DNA alterations for all 30 cases by chromosome arm is shown in Table 2 (with LOH) and Additional file 5/Table S3 (without LOH). CNNLOH was observed on all chromosome arms, but most frequently on 19p (100%), 5p (96%), 2p (95%), and 20q (95%). The highest frequencies of LOH with CN loss (CN = 1) were found on 3p (56%), 5q (47%), and 21q (41%); relatively high frequencies were also seen on 18q (31%), 11q (29%), 1p (28%), 19q (27%), and 11p (25%). LOH with CN gain was most common on 20p (82%), 8q (74%) and 3q (42%) (Table 2 and Figure 1). Taken together, our results show that LOH with CNN or CN gain were much more frequent than LOH with CN loss on every chromosome arm but one (ie, 3p).

**Table 2:** LOH by copy number in ESCC cases by chromosomal arm (N = 30 cases)

Chromosomal arm	Total no. informative SNPs with LOH	No. informative SNPs with LOH and CN = 1 (fraction)	No. informative SNPs with LOH and CN = 2 (fraction)	No. informative SNPs with LOH and CN = 3 or 4 (fraction)
1p	10,576	2,930 (0.28)	6,522 (0.62)	1,124 (0.11)
1q	8,366	459 (0.05)	7,674 (0.92)	233 (0.03)
2p	11,321	371 (0.03)	10,770 (0.95)	180 (0.02)
2q	23,115	3,015 (0.13)	18,939 (0.82)	1,161 (0.05)
3p	27,593	15,335 (0.56)	12,064 (0.44)	194 (0.01)
3q	7,256	41 (0.01)	4,168 (0.57)	3,047 (0.42)
4p	12,223	4,771 (0.39)	7,452 (0.61)	0 (0)
4q	28,105	9,142 (0.33)	17,905 (0.64)	1,958 (0.04)
5p	1,315	0 (0)	1,259 (0.96)	56 (0.04)
5q	16,537	7,744 (0.47)	8,777 (0.53)	16 (0)
6p	5,622	342 (0.06)	4,870 (0.87)	410 (0.07)
6q	3,365	165 (0.05)	2,773 (0.82)	427 (0.13)
7p	7,389	9 (0)	5,491 (0.74)	1,889 (0.26)
7q	8,001	151 (0.02)	6,452 (0.74)	1,398 (0.17)
8p	8,803	1,580 (0.18)	6,234 (0.71)	989 (0.11)
8q	17,321	47 (0)	4,633 (0.27)	12,641 (0.74)
9p	18,292	2,772 (0.15)	13,333 (0.73)	2,187 (0.12)
9q	31,400	1,965 (0.06)	27,212 (0.87)	2,223 (0.07)
10p	2,739	472 (0.17)	2,063 (0.75)	204 (0.07)
10q	14,651	1,728 (0.12)	12,075 (0.82)	848 (0.06)
11p	9,391	2,367 (0.25)	6,902 (0.73)	122 (0.01)
11q	17,377	5,116 (0.29)	10,422 (0.60)	1,839 (0.11)
12p	5,229	156 (0.03)	3,251 (0.62)	1,822 (0.34)
12q	6,794	67 (0.01)	5,980 (0.88)	747 (0.11)
13q	35,648	5,897 (0.17)	25,964 (0.73)	3,787 (0.11)
14q	10,931	1,484 (0.14)	7,446 (0.68)	2,001 (0.18)
15q	10,194	685 (0.07)	8,313 (0.82)	1,196 (0.12)
16p	1,395	111 (0.08)	1,284 (0.92)	0 (0)
16q	3,127	1 (0)	2,832 (0.91)	294 (0.09)
17p	7,719	324 (0.04)	6,939 (0.90)	456 (0.06)
17q	16,328	38 (0)	14,754 (0.90)	1,536 (0.09)
18p	2,596	488 (0.19)	1,147 (0.44)	961 (0.37)
18q	9,978	3,108 (0.31)	6,838 (0.69)	32 (0)
19p	1,069	1 (0)	1,065 (1.00)	3 (0)
19q	3,505	932 (0.27)	2,482 (0.71)	91 (0.03)
20p	1,278	0 (0)	229 (0.18)	1,049 (0.82)
20q	1,773	60 (0.03)	1,677 (0.95)	36 (0.02)
21q	9,444	3,825 (0.41)	5,599 (0.59)	20 (0)
22q	4,177	621 (0.15)	3,274 (0.80)	222 (0.05)

Range (fraction SNPs with LOH)		0-0.56	0.18-1.00	0-0.82
Median (fraction SNPs with LOH)		0.05	0.71	0.05
Global average (fraction SNPs with LOH)		0.19	0.70	0.11

Results of CN alterations in non-LOH group by chromosome arms are summarized in Additional file 5/Table S3. Briefly, a frequency of CN loss ≥ 10% was observed on eight chromosome arms (3p, 4p, 4q, 5q, 8p, 9p, 11q, and 13q). A frequency of CN gain ≥ 10% was observed on 13 chromosome arms (1q, 2p, 2q 3q, 5p, 7p, 7q, 8q, 12p, 14q, 18p, 20p, and 20q).

### Relation between genomic alterations and gene expression

The average present call rate on the Human Genome U133A array was 53% (range 51- 61%) for the 34 chips from the 17 sample pairs with sufficient tissue for RNA isolation and testing. To investigate the relation between LOH/CNV and gene expression levels, we intersected genes on the Affymetrix U133A chip with SNPs on the 500 K SNP array. SNPs that mapped within genes are summarized in Additional file 6/Table S4 and include 169,687 SNPs within 12,225 genes.

We were interested in identifying differentially-expressed genes between LOH and non-LOH groups in genes that were CN neutral. A total of 4,572 genes qualified for this analysis (see Methods). Among these genes, 168 genes showed significant differences in expression between tumors with and without LOH (*P *< 0.01) (Additional file 7/Table S5). Based on chance alone (at the *P *< 0.01 level), differences in only 45 genes would be expected, therefore, expression differences were observed in over three times as many genes as expected. One hundred and one (60%) of the 168 genes showed lower expression levels in CNNLOH than in the normal group (ie, CNN, no LOH), whereas 67 genes (40%) showed higher expression levels in CNNLOH (Additional file 7/Table S5). Twenty-eight of the 101 down-regulated genes (32 probes) and 18 of the 67 up-regulated genes (19 probes) showed expression differences ≥ 2-fold (Table 3). These findings suggest that in the CN neutral state, LOH can affect gene expression.

**Table 3:** Comparison of gene expression in copy number neutral (CNN) genes with LOH and without LOH (normal) (N = 46 genes significantly differentially-expressed 2-fold or greater)*

Gene name	Cytoband	Probeset on U133A array	No. cases with LOH	No. cases without LOH	No. SNPs	Fold change CNN with LOH	Fold change CNN without LOH	T-test	P-value
									
***Genes where expression level of CNN with LOH is less than CNN without LOH (N = 28 genes)***									
*ADK*	chr10q22|10q11-q24	204120_s_at	2	13	46	0.404	0.606	3.138	8.559E-03
*ADK*	chr10q22|10q11-q24	204119_s_at	2	13	46	0.411	0.619	3.195	7.278E-03
*AIM1L*	chr1p36.11	220289_s_at	2	15	6	0.093	0.201	3.548	4.812E-03
*APOL6*	chr22q12.3	219716_at	2	5	4	0.467	0.894	4.659	9.543E-03
*ATP6V0E1*	chr5q35.2	201171_at	2	12	5	0.375	0.669	3.234	9.843E-03
*ATP6V0E1*	chr5q35.2	200096_s_at	2	12	5	0.480	0.872	4.445	5.699E-03
*BACH1*	chr21q22.11	204194_at	2	12	9	0.498	0.738	3.970	2.098E-03
*BTG2*	chr1q32	201236_s_at	2	14	1	0.460	0.986	4.095	1.183E-03
*CD2*	chr1p13.1	205831_at	2	14	1	0.324	0.628	3.462	9.221E-03
*CEACAM6*	chr19q13.2	211657_at	2	13	3	0.007	0.100	4.779	7.023E-03
*CRAT*	chr9q34.1	209522_s_at	7	8	1	0.293	0.567	3.169	7.495E-03
*CSDE1*	chr1p22	202646_s_at	2	14	3	0.343	0.895	7.835	7.590E-05
*CYP4F3*	chr19p13.2	206515_at	2	13	136	0.065	0.307	3.329	5.631E-03
*DIO2*	chr14q24.2-q24.3	203699_s_at	2	11	4	0.072	0.127	3.565	6.175E-03
*DIO2*	chr14q24.2-q24.3	203700_s_at	2	11	4	0.168	0.280	3.697	3.752E-03
*EIF4EBP2*	chr10q21-q22	208770_s_at	2	14	1	0.452	0.657	6.300	2.692E-05
*FAS*	chr10q24.1	216252_x_at	2	13	8	0.412	0.648	3.058	9.883E-03
*HERC6*	chr4q22.1	219352_at	3	9	1	0.105	0.538	5.769	2.901E-04
*ICAM3*	chr19p13.3-p13.2	204949_at	2	13	2	0.356	0.602	3.478	4.342E-03
*IL1RN*	chr2q14.2	212659_s_at	2	12	13	0.073	0.142	3.256	7.793E-03
*NADSYN1*	chr11q13.4	218840_s_at	2	8	10	0.448	0.649	3.399	9.384E-03
*NHP2*	chr5q35.3	216583_x_at	2	10	1	0.497	0.774	4.091	2.584E-03
*PDCD4*	chr10q24	202730_s_at	2	12	4	0.212	0.363	4.483	9.256E-04
*PPP1R13L*	chr19q13.32	218849_s_at	3	13	1	0.190	0.487	5.016	5.018E-04
*RIPK4*	chr21q22.3	221215_s_at	5	10	7	0.217	0.454	3.614	3.658E-03
*SKAP2*	chr7p21-p15	216899_s_at	2	10	12	0.293	0.615	4.165	1.973E-03
*SKAP2*	chr7p21-p15	204361_s_at	2	10	43	0.359	0.665	4.384	1.591E-03
*SLC6A1*	chr3p25-p24	205152_at	2	8	6	0.158	0.438	5.044	2.481E-03
*STK39*	chr2q24.3	202786_at	2	12	86	0.151	0.290	3.363	6.327E-03
*SYNPO2L*	chr10q22.2	219804_at	3	13	1	0.063	0.345	6.925	8.703E-06
*TGM5*	chr15q15.2	207911_s_at	2	13	8	0.382	0.517	3.496	4.389E-03
*ZNF91*	chr19p13.1-p12	206059_at	2	10	5	0.026	0.551	9.054	6.430E-04

									
***Genes where expression level of CNN with LOH is greater than CNN without LOH (N = 18 genes)***									
*ACVR1*	chr2q23-q24	203935_at	2	12	13	3.480	1.751	-4.533	7.869E-04
*ASPN*	chr9q22	219087_at	5	10	2	10.594	2.679	-3.792	2.667E-03
*BUB1*	chr2q14	209642_at	2	12	2	8.571	4.010	-3.922	2.170E-03
*CALU*	chr7q32.1	200757_s_at	2	12	7	3.467	1.830	-4.612	7.505E-04
*CALU*	chr7q32.1	200755_s_at	2	12	7	3.899	1.981	-3.903	2.269E-03
*CAMSAP1L1*	chr1q32.1	212765_at	2	13	3	3.000	1.357	-6.288	1.629E-03
*CENPF*	chr1q32-q41	209172_s_at	2	14	10	5.481	2.643	-5.909	3.926E-05
*CSNK1E*	chr22q13.1	202332_at	2	3	4	2.933	1.157	-7.434	9.278E-03
*FAM13A*	chr4q22.1	202973_x_at	3	10	14	2.756	1.149	-3.508	6.115E-03
*FHL2*	chr2q12-q14	202949_s_at	2	14	15	3.159	1.244	-4.697	4.097E-04
*GSR*	chr8p21.1	205770_at	3	10	8	2.505	1.222	-3.701	4.824E-03
*ITGA6*	chr2q31.1	201656_at	2	11	21	4.038	2.400	-3.519	4.820E-03
*KIF14*	chr1q32.1	206364_at	2	13	6	11.027	3.578	-8.257	2.071E-06
*NCAPH*	chr2q11.2	212949_at	2	11	2	3.643	1.943	-5.087	4.450E-04
*NRP2*	chr2q33.3	214632_at	3	11	29	2.161	1.257	-3.500	9.276E-03
*RBM28*	chr7q32.1	218593_at	2	11	9	2.210	1.555	-3.976	2.370E-03
*SMAD5*	chr5q31	205187_at	2	12	3	2.208	0.829	-10.692	3.171E-07
*STEAP3*	chr2q14.2	218424_s_at	2	14	12	2.707	1.207	-6.922	9.985E-06
*TGS1*	chr8q11	219231_at	2	8	1	2.533	1.656	-3.495	8.601E-03

*Sorted alphabetically by gene name

We also compared expression of genes with LOH versus no LOH in CN loss genes. We identified six of 600 genes which showed significantly different expression between the LOH groups. All six genes showed increased expression in tumors with LOH (Table 4a).

**Table 4:** Comparison of gene expression in copy number loss/gain genes with LOH and without LOH*

Gene name	Cytoband	Probeset on U133A array	No. cases with LOH	No. cases without LOH	No. SNPs	Fold change with LOH	Fold change without LOH	T-test	P-value
									
***Table 4.a.: Genes/probes with CN loss (CN = 1)***									
*ATXN7*	chr3p21.1-p12	204516_at	4	2	10	0.574	0.258	-8.233	1.333E-03
*CTDSPL*	chr3p21.3	213597_s_at	3	3	13	0.997	0.890	-5.414	8.188E-03
*F2R*	chr5q13	203989_x_at	3	2	4	6.083	1.217	-6.771	7.275E-03
*RAB5A*	chr3p24-p22	209089_at	5	2	6	0.419	0.240	-9.065	5.698E-04
*RAF1*	chr3p25	201244_s_at	3	2	9	0.555	0.389	-24.008	5.074E-04
*SCN10A*	chr3p22-p21	208578_at	3	4	23	1.806	0.884	-4.253	8.107E-03

									
***Table 4.b.: Genes/probes with CN gain (CN = 3 or 4)***									
*ARHGAP5*	chr14q12	217936_at	2	5	1	1.731	0.769	-4.809	8.159E-03
*CYP11B1*	chr8q21	214610_at	2	6	3	0.915	1.114	4.728	3.469E-03
*NFATC4*	chr14q11.2	205897_at	2	3	1	1.363	0.903	-11.692	2.823E-03
*PAX9*	chr14q12-q13	207059_at	2	5	1	0.898	0.319	-5.395	5.706E-03
*PPFIA1*	chr11q13.3	202066_at	7	2	12	7.524	2.448	-3.918	6.425E-03
*TP63*	chr3q28	211834_s_at	2	13	17	0.901	1.197	3.438	9.809E-03
*TP63*	chr3q28	211194_s_at	2	13	18	0.987	2.649	4.008	3.640E-03

*Sorted alphabetically by gene name

Finally, we compared gene expression in the CN gain state between tumors with and without LOH. We found that six of 354 genes showed significant differences in expression between the two groups, including two down-regulated and four up-regulated genes (Table 4b).

## Discussion

We characterized ESCC tumors for complex DNA alterations - LOH and CNV - and related these genomic alterations to gene expression. To our knowledge, this is the first report to comprehensively address the distribution of complex DNA alterations in ESCC and its relation to gene expression on a genome-wide scale.

Ninety percent of cases showed CNNLOH in their tumors and, over all cases, CNNLOH was found on every chromosome arm, indicating that it is a common phenomenon.

The frequency of CNNLOH observed here in ESCC was much less than has been reported in other cancers [3-19]. For example, in colon cancer and basal cell carcinoma nearly all LOH was associated with copy number neutral regions [3,10]. In general, CNNLOH occurs with variable frequency in different genomic regions in tumors of different origin. There are several differences between the study reported here and previous studies which likely influenced the results. First, DNA from micro-dissected tumor and adjacent normal was used in the present study, while either cancer DNA without matched controls or cancer cell lines were used in most other reported studies. Second, we examined LOH and CN alterations using the same SNP array platform, while other studies used SNPs for LOH and CGH arrays for CN analyses. Third, the criteria for identifying LOH differed among the studies reported. Finally, the types of cancers studied previously differ from the present study which is the first report of CNNLOH in ESCC.

In previous LOH studies, we reported high-frequency LOH on several chromosome arms, including 3p, 4p, 4q, 9p, 9q, 13q, 17p, and 17q [23,26,27]. By integrating LOH and CN alteration data in the present study, we can now say that the LOH on 3p is primarily due to CN loss LOH, while the LOH on the other seven chromosome arms is predominantly due to CNNLOH.

Our results showed that CNNLOH can change expression levels of genes in ESCC, either increasing or decreasing them. We do not know why CNNLOH changes gene expression, but one possibility is that the two alleles may have different gene expression levels. For example, if allele A expression is greater than allele B, the expression level for the 3 genotypes would be ordered as AA > AB > BB. CNNLOH with retention of two B alleles (genotype BB) would then show lower expression than genotype AB. Conversely, CNNLOH with loss of the allele B would result in two copies of allele A and a higher level of expression than that of AB cells. Another possibility is that the two alleles have different expression due to different epigenetic states, with LOH resulting in copies with two extreme epigenetic states. A third possibility is that one allele harbors a mutation and subsequent LOH leads to a homozygous mutant. Several studies have shown that CNNLOH regions can harbor mutated genes. For example, *JAK2 *V617F, *FLT3-ITD*, *AML1/RUNX1*, *WT1*, and *NPM1 *mutations were all found in CNNLOH regions in AML [15]. These various hypotheses merit testing in the future.

The study design in the present study has several important features: (i) we compared CN status between DNA from germ-line and micro-dissected adjacent normal tissue; (ii) we used micro-dissected DNA from tumor tissue; (iii) we assessed both LOH and CN alterations simultaneously using the same array platform; and (iv) we integrated complex DNA alterations and gene expression data on a genome-wide level using both high density SNP and expression arrays in the same cases. A noteworthy weakness of our study is the relatively small number of cases evaluated (including a particularly small number of cases with both LOH and RNA expression data to evaluate, due in part to the 500K chip mean heterozygosity of 27%), which limited our power to detect significant differences in loci between LOH and non-LOH groups. In addition, findings for ESCC from this high-risk region may not be generalizable to populations elsewhere in the world.

In summary, we investigated the distribution of complex DNA alterations in ESCCs at the genome-wide level and determined that CN neutral is the most common CN state in LOH, and that CNNLOH is a very common phenomenon overall. Importantly, we also showed that CNNLOH could alter the expression level of genes affected in ESCC.

## Conclusion

CNNLOH is a common phenomenon in many cancers, including ESCC, and non-disjunction and/or somatic recombination are the most likely mechanisms for its occurrence. CNNLOH can result in changes in gene expression which are functionally significant. Expression differences in CNNLOH suggest that alleles are different in terms of their gene expression potential, and that these differences may result from differences in genotype and/or epigenetics.

## Abbreviations

SNP: single nucleotide polymorphism; ESCC: esophageal squamous cell carcinoma; (CNNLOH): copy number neutral loss of heterozygosity; CN: copy number.

## Authors' contributions

NH, AMG, TD, and PRT designed, conducted, and supervised the field and clinical studies; PRT obtained funding for the project; NH and CW designed and performed the laboratory analyses; NH, RJC, HHY, and MPL conducted the statistical analyses; NH and ML drafted the manuscript; NH, RJC, AMG, PRT, and MPL conceptualized the data analyses and revised and edited the manuscript. All authors read and approved the final manuscript.

## Supplementary Material

Additional file 1 **Figure S1**. Definition of LOH/CN blocks within a single sample. Click here for file

Additional file 2 **Figure S2**. The relationship between genes, Affymetrix expression probesets, and SNPs. Click here for file

Additional file 3 **Table S1**. Comparison of copy number alterations between DNA from blood and microdissected normal tissue. Click here for file

Additional file 4 **Table S2**. Copy number status of SNPs without LOH by case (N = 30). Click here for file

Additional file 5 **Table S3**. CN status of SNPs without LOH by chromosome arm (N = 30 cases). Click here for file

Additional file 6 **Table S4**. No. of genes matched on Affymetrix 500 K SNP and U133A expression arrays. Click here for file

Additional file 7 **Table S5**. Comparison of gene expression in copy number neutral (CNN) genes with LOH and without LOH (normal) (N = 168 genes significantly differentially-expressed). Click here for file
