# Supplementary material for: Genome wide analysis of DNA copy number neutral loss of heterozygosity (CNNLOH) and its relation to gene expression in esophageal squamous cell carcinoma
Source: BMC Genomics. 2010 Oct 18;11:576. doi: 10.1186/1471-2164-11-576 (PMC3091724; doi:10.1186/1471-2164-11-576)
Supplement: Additional file 2 — Figure S2. The relationship between genes, Affymetrix expression probesets, and SNPs. [file 1471-2164-11-576-S2.PDF]

Supplementary Figure 2:
The relationship between genes, Affymetrix expression probesets and SNPs

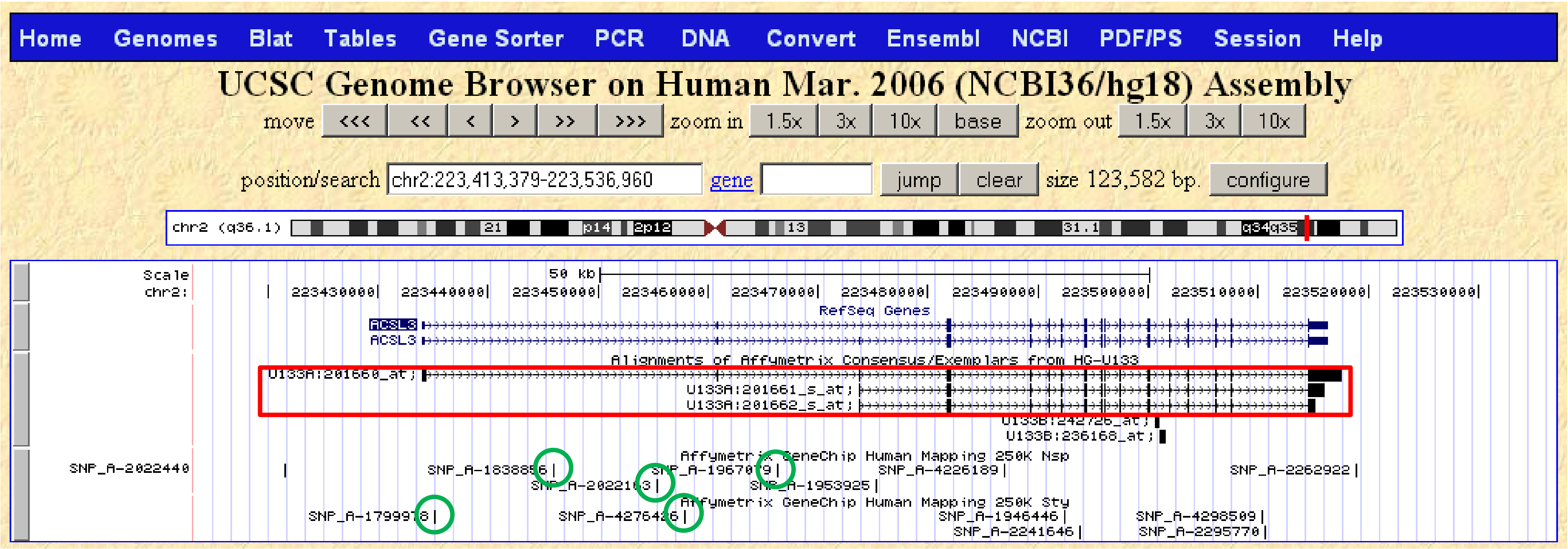

All features are mapped onto the *hg18* reference sequence.

The ACSL3 gene transcript is shown in blue.

Reference sequences for 3 Affymetrix U133A probesets designed against the gene are boxed in red.

Five SNPs encompassed only by the reference sequence for probeset 201660_at are circled in green.

Data from: http://genome.ucsc.edu/
